# Consequences of Chronic Non-Cancer Pain in adulthood. Scoping Review.

**DOI:** 10.11606/s1518-8787.2020054001675

**Published:** 2020-04-03

**Authors:** Rocío Cáceres-Matos, Eugenia Gil-García, Sergio Barrientos-Trigo, Ana María Porcel-Gálvez, Andrés Cabrera-León

**Affiliations:** I Universidad de Sevilla Facultad de Enfermería, Fisioterapia y Podología Departamento de Enfermería Sevilla España Universidad de Sevilla. Facultad de Enfermería, Fisioterapia y Podología. Departamento de Enfermería. Sevilla, España; II Escuela Andaluza de Salud Pública Granada España Escuela Andaluza de Salud Pública. Granada, España; III Centro de Investigación Biomédica en Red de Salud Pública y Epidemiología Madrid España Centro de Investigación Biomédica en Red de Salud Pública y Epidemiología (CIBERESP). Madrid, España; IV Universidad de Granada Instituto de Investigación Biosanitaria Hospitales Universitarios de Granada Granada España Universidad de Granada. Instituto de Investigación Biosanitaria, ibs. Hospitales Universitarios de Granada. Granada, España

**Keywords:** Adult, Adulthood, Chronic Pain, Cost of disease, Daily Activities, Quality of life, Review

## Abstract

**OBJECTIVE:**

To examine and map the consequences of chronic pain in adulthood.

**METHOD:**

Documents addressing the impact of chronic pain on the psychological and social spheres of people suffering from chronic pain, published in Spanish and English between 2013 and 2018, were included. Those who addressed pharmacological treatments, chronic pain resulting from surgical interventions or who did not have access to the full text were excluded. Finally, 28 documents from the 485 reviewed were included

**RESULTS:**

Studies show that pain is related to high rates of limitation in daily activities, sleep disorders and anxiety-depression spectrum disorders. People in pain have more problems to get the workday done and to maintain social relationships. Chronic pain is also associated with worse family functioning.

**CONCLUSIONS:**

This review shows that limitations in the ability to perform activities of daily living, sleep, psychological health, social and work resources and family functioning are lines of interest in published articles. However, knowledge gaps are detected in areas such as the influence of having suffered pain in childhood or adolescence, the consequences of non-fulfillment of working hours and gender inequalities.

## INTRODUCTION

Pain is an unpleasant sensory and emotional experience that acts as a sign of biological alertness in the face of real or potential tissue damage^1^ . It can be classified according to etiology, anatomical location or duration^2^ . According to the latter, chronic non-cancer pain (CNCP) persists continuously or intermittently for a period of more than three months and is not associated with cancer processes^3^ .

CNCP is considered a public health problem that affects 20% to 35% of the world’s population^4^ , 19% of the European population^5^ and 17% of the Spanish population^6^ . When pain persists for long periods of time, it loses its protective purpose^7^ and becomes the result of a complex process in which biological, psychological and sociocultural^8^ factors interact with each other^9^ . Several international organizations, such as the European Pain Federation or the International Association for the Study of Pain, consider that a change of perspective in the assessment and treatment of pain is necessary, being addressed as an entity in itself^10^ , in which the family, social and cultural context that the person lives is considered^11^ .

In the last ten years, scientific production on the repercussions of pain has increased. Numerous studies state that the perception and appreciation of CNCP affect sleep^12^ or limitations of daily activity^13^ , but also psychological and social factors such as anxiety, depression^14^ , self-esteem^15^ , coping^16^ , resilience^13^ , social and family support^7^ and/or use of toxic substances^14^ .

Therefore, we consider it necessary to know the individual and social consequences that CNCP has on the adult population. For this, we conducted a Scoping Review of the scientific literature. Our goal is to examine and map the consequences of chronic pain in adulthood.

## METHODS

The Scoping Review methodology is used to map the scientific literature and detect areas of study that are not sufficiently researched^16^ . To accomplish this, we used the methodological framework proposed by Arksey and O’Malley^17^ and the Joanna Briggs Institute Reviewer’s Manual^18^ . Although the Scoping Review methodology does not require quality assessment, several authors consider it a strength^19^ , as it allows to make recommendations for clinical practice^20^ .

In view of these considerations, we assessed the level of evidence (LE) and the degree of recommendation (DR) of the studies included in accordance with the Scottish Intercollegiate Guidelines Network^21^ . In this case, LE and DR scores are related. Thus, for an investigation with NE 1++; 1+ or 1- is considered extremely recommended (DR A); for NE 2++, 2+ or 2- the recommendation rating would be favorable (DR B), and for LE 3 and 4 the degrees of recommendation would be favorable, but not conclusive (DR C), and it is not recommended or disapproved (DR D), respectively.

### Databases and research strategy

The search in the literature was performed in four scientific databases (CINAHL, PubMed, Scopus and Web of Science) and in gray literature deposits (Research Repository of the University of Seville IDUS, TESEO, Openthesis, Opengrey, Grey Literature Report, American Pain Society, European Pain Federation, and International Association for the Study of Pain).

They were held between November and December 2018 with the following search strategy: (“chronic pain” OR “persistent pain” OR “long term pain”) and (adult*) AND (impact* OR influenc* OR cause* OR outcome* OR result* OR consequence* OR effect* OR repercussion*).

As inclusion criteria, documents addressing the biopsychosocial consequences of chronic non-cancer pain were considered: 1) documents addressing the biopsychosocial consequences of chronic non-cancer pain; (2) in adults between 18 and 65 years old, not hospitalized; 3) published between 2013 and December 2018 and 4) written in Spanish or English.

Exclusion criteria were: 1) documents dealing with pharmacological treatments; 2) documents that address people with chronic non-cancer pain resulting from neurodegenerative diseases, chronic infectious diseases or cognitive impairment and 3) documents that did not have access to the full text.

### Screening

After the removal of duplicate articles in the research, the selection process was performed, evaluating the relevance of the studies identified. This process was performed by two reviewers independently.

In the first phase, reviewers assessed the titles and abstracts found to exclude articles that did not meet the inclusion and exclusion criteria. Later, they reviewed their contents in full text and decided which ones were included in the data extraction phase. In case of disagreement about the inclusion of an article between the two reviewers, it was discussed with the rest of the research team until consensus was reached.

### Data extraction and analysis

Team members produced a document that served as a model for the extraction of information from the article to be standardized. In addition, they developed a protocol to systematize the work procedure. Two of the team members independently collected information about each of the articles (authors’ names, year and country of publication, study objective and methodology, sample size and characteristics, intervention, main results obtained and key points). All team members discussed the information in cases of disagreement until consensus was reached.

## RESULTS

After the review, 485 articles whose titles met the inclusion criteria were obtained. The search for gray literature produced 60 documents. Once duplicates were removed, 285 titles and abstracts were reviewed using the inclusion and exclusion criteria. Exactly 170 full texts were recovered for screening. After reviewing the full texts, 142 were excluded, because they did not report the theme of the study, remaining 28 documents for analysis ( Figure ). The studies included were classified into four areas based on the effects of chronic pain: activities of daily living (ADL); sleep (S); psychological health (PH) and socio-labor and family consequences (SLFC) ( Table ).

**Figure:**
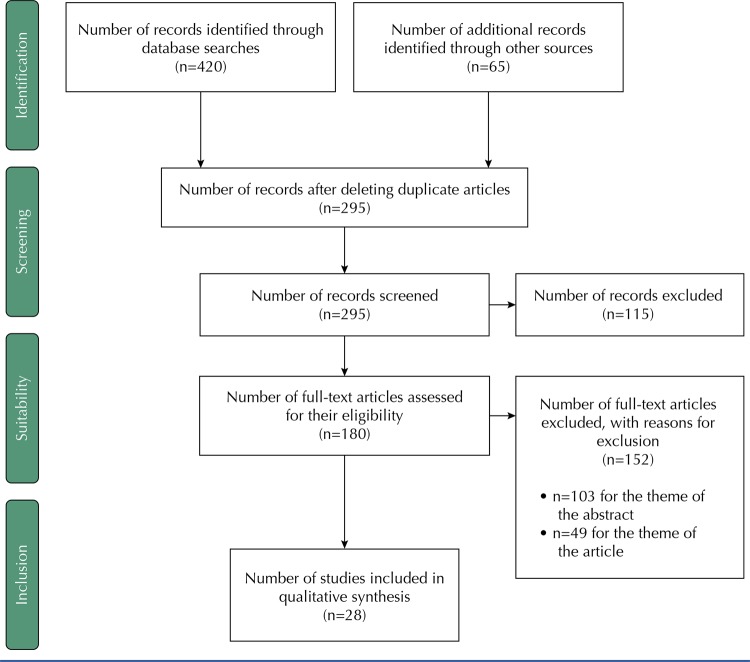
Consequences of chronic non-cancer pain in adulthood. Scoping Review.

**Table:** Main characteristics of the studies included in the review.

Authors, year, country	Objective	Methodology/sample	Intervention	Results	Key points	LE	DR
Altug et al. 2015 Turkey	Compare pain, emotional state and disability level in patients with chronic cervical pain and low back pain.	Cross-sectional study. N=100 patients	Visual Analogue Scale (VAS) Owestry Disability Index (ODI) Neck Disability Index (NDI) Beck Depression Inventory (BDI)	No significant difference was found for the intensity and duration of pain between the low back pain group and the group with neck pain. Pain duration, emotional state and disability level in patients with low back pain were worse than in patients with neck pain.	S, PH	4	C
Akbari et al. 2016 Iran	Examines the role of individual and family variables in understanding chronic pain related to disability.	Cross-sectional study. N=142 people	Visual Analogue Scale (VAS) Tampa Scale of Kinesiophobia (TSK) Pain Catastrophizing Scale (PCS) Roland Morris Disability Questionnaire (RDQ) Anxiety Stress Scales (DASS) Family Assessment Device (FAD)	Family dysfunction can contribute to catastrophic thinking, which in turn contributes to the inability of patients, increasing the fear of movement and depression.	S, PH, SLFC	4	C
Andrews et al. 2014 Australia	Examine the association between daily physical activity and sleep in people with chronic pain.	Prospective observational study. N=50 people	Demographic questionnaire Visual Analogue Scale (VAS)	Individuals who claimed to have a greater number of pain points had longer periods of vigil at night.	ADL	4	C
Bailly et al. 2015 France	Understand the experiences of patients living with low back pain. Monocentric qualitative study.	Monocentric qualitative study. N=25 people (11 men and 14 women)	Semi-structured interview and focus groups	The participants reported negative perception in social interactions, with shame and frustration in relation to their difficulties in performing daily activities. They felt misunderstood and unsupported due to the absence of visible signs of the condition.	PH	5	D
Boggero et al. 2015 USA	Test independent and interactive contributions of the somatosensory component of pain and affective component of pain in emotional, social and daily functioning.	Cross-sectional study N=472 people	The West Haven-Yale Multidimensional Pain Inventory (MPI) Visual Analog Scale (VAS)	Women showed higher overall activity than males. Higher levels of pain resulted in worse results in all areas of functioning.	S, PH	4	C
Cabrera-Perona et al. 2017 Spain	Evaluate the association between social comparison, catabolism and specific health outcomes.	Cross-sectional study. N=131 people		A positive correlation was found between catabolism and functional deficiency, psychological distress, anxiety and depression in fibromyalgia.	S, PH	4	C
Calandre et al. 2015 Spain	Evaluate the prevalence of suicidal ideation among a sample of patients with fibromyalgia.	Cross-sectional study. N=373 people	Fibromyalgia Impact Questionnaire The Beck Depression Inventory (BDI) The Pittsburgh Sleep Quality Index The Brief Pain Inventory (BPI) SF-12 Health Survey	48% of people with chronic pain reported having suicidal thoughts and 31 percent attempted suicide. There were no differences in the age or duration of the disease among patients without suicidal ideation, suicidal thinking and suicidal behavior.	S	4	C
Campbell et al. 2013 UK	Proving that sleep problems in people with chronic pain increase the risk of initiating depression.	Prospective cohort study. N=2,622 people	Jenkins Sleep Questionnaire Hospital Anxiety and Depression Scale (HADS)	Sleep problems can lead to depression three years after it begins. Treatments may include preventing or treating sleep problems, as well as focusing on pain treatment.	ADL, S	1b	A
Campbell et al. 2015 New Zealand	Document the prevalence and correlations of chronic pain and suicide, estimating the contribution of chronic disease to suicide.	Population study. N=8,841 people.	Australian National Mental Health Survey.	The chances of suicide were two to three times higher in people in pain. Sixty-five percent of people who have tried to kill themselves in the last 12 months had a history of chronic pain.	S	3b	B
Castro et al. 2014 Spain	Evaluate sleep quality and the prevalence of severe depressive disorder and generalized anxiety disorder in patients with chronic musculoskeletal pain.	Cross-sectional study. N=39 people	International Neuropsychiatric Interview. Pittsburgh Questionnaire Montgomery-Asberg Depression Scale	69.2% of the sample had severe depressive disorder and/or 65.5% had generalized anxiety disorder. No statistically significant differences in sleep quality were found between groups treated with potent opioids and treated with smaller opioids/anti-inflammatory drugs.	ADL, S	4	C
Danya et al. 2015 France	Explore the relationship between time perspective and psychological difficulties.	Cross-sectional study. N=264 people	Zimbardo Time Perspective Inventory (ZTPI) Pain Beliefs and Perceptions Inventory (PBPI) Hospital Anxiety and Depression Scale (HADS)	Patients with children were more depressed, those at the educational level were more anxious, and those who were unemployed during the disease had higher levels of depression.	S, PH	4	C
De Sola et al. 2017 Spain	Determine prevalence and factors related to medical leave and loss of employment among people suffering from chronic pain.	Cross-sectional study. N=1,543 people	Telephone survey	The prevalence of medical leave due to CP in the Spanish population was 4.21%, being more prevalent in people who need help dressing and caring, taking medications and/or with higher schooling. The prevalence of job loss was 1.8%.	PH, SLFC	4	C
Ditre et al. 2013 USA	Evaluate the importance of pain-related anxiety in relation to tobacco dependence in a sample of smokers with chronic pain.	Cross-sectional study. N=129 people	Pain Anxiety Symptoms Scale (PASS-20) Wisconsin Inventory of Smoking Dependence Motives (WISDM) Chronic Pain Screener Graded Chronic Pain Scale (GCPS) Generalized Anxiety Disorder (GAD-7) Fagerström Test for Nicotine Dependence (FTND)	Pain-related anxiety was strongly associated with tobacco dependence. Smokers may be at risk of maintaining and exacerbating their dependence on tobacco, possibly due to individual differences in pain-related anxiety.	S	4	C
Ferreira-Valente et al. 2014 Portugal	Evaluate coping responses, beliefs, and social support to adapt positively.	Observational study. N=324 people	Numerical Rating Scale (NRS) Portuguese Brief Pain Inventory Interference Scale (P-BPI) Physical Component Summary (MCS) Portuguese Pain Self-Efficacy Questionnaire (P-PSEQ) Social Support Satisfaction Scale (ESSS) Portuguese 2-item per Scale Coping Strategies Questionnaire (CSQ-14)	Coping responses and perceived social support were related to interference in pain and psychological functioning. Coping responses were positively associated with pain interference and negatively to physical and psychological functioning.	ADL, S, PH	4	C
Henne et al. 2015 Australia	Explore the relationship between persistent pain, anguish, and emotional connection.	Cross-sectional study. N=388 women	The McGill Pain Questionnaire (SF-MPQ) Depression, anxiety and stress (DASS-21– short form)	Many women with chronic pain have difficulties in emotional connection that attribute to the experience of persistent pain.	S	4	C
Inoue et al. 2015 Japan	Estimate the prevalence of chronic pain in Japan, analyze the associated factors and evaluate social burden due to chronic pain.	Cross-sectional study N=6,000 people	11-point Numerical Rating Scale (NRS)	Chronic pain worsens with environmental factors. People who exercise had lower rates of pain. Women suffered more pain than men. Elderly, living alone and unemployed were also associated with chronic pain.	PH	4	C
Jensen et al. 2015 Denmark	Describe the associations between demographics and health-related quality of life of patients with chronic non-cancer pain.	Cohort study N=1,176 people	Hospital Anxiety and Depression Scale (HADS) Short-Form 36 Bodily Pain Scale (SF-36).	Patients with chronic pain scored less in all domains, especially in physical activity, pain level and vitality. 75% of patients assess that their physical health affects their work and/or daily activity.	PH	1b	A
Karaman et al. 2014 Turkey	Examine the prevalence and relationship between sleep disorders and chronic pain.	Observational study. N=2,635 people	Visual Analogue Scale (VAS) Pain Sleep Quality Index (PSQI)	33% of patients with chronic pain between 20 and 30 years and 47.8% between 60 and 70 years presented worse sleep quality.	ADL	4	C
Mun et al. 2017 USA	Test the effect of pain expectancy with pain after pain.	Cross-sectional study. N= 451 people	Filling out a pain diary.	Pain management today predicts decreased pain the next day. Negativity and coping are not related to the level of pain the next day.	PH	4	C
Nicholas et al. 2017 USA	Examine the interaction between pain and short-term physical functioning.	Longitudinal study. N=389 people	15-item Brief Pain Inventory 2-item Short-Form 36 Bodily Pain Scale (SF-36)	Therapies to control the consequences of chronic pain in relevant aspects of life and physical activities are necessary for restoration of vital activity.	PH	2b	B
Orhurhu et al. 2015 USA	Determine the prevalence of smoking in patients with chronic pain.	Population study N=5,350 patients		The prevalence of smoking in adults with chronic pain was 23.5%.	S	4	C
Raijmakers et al. 2015 Alemannia	Determine differences in the level of daily activity between people with and without pain.	Clinical trial N=37 people		Patients with chronic pain have a lower overall level of physical activity than the healthy population.	PH	1b	A
Stefane et al. 2013 Brazil	Evaluate perceived pain, disability and quality of life in individuals with chronic low back pain.	Cross-sectional study. N=97 people	Roland Morris Questionnaire (RMQ) WHOQOL-Brief	The perceived score of pain intensity, disability and quality of life were the most affected.	ADL, SP	4	C
Triñanes et al. 2014 Spain	Describe the relationship of suicidal ideation in fibromyalgia.	Cross-sectional study. N=117 women	Beck Depression Inventory (BDI) The Pittsburgh Sleep Quality Index (PSQI) The Fibromyalgia Impact Questionnaire (FIQ) Short-Form 36 Health Survey (SF-36) Visual Analogue Scale (VAS)	Patients with fibromyalgia have a moderate level of depression, sleep dysfunction and severe deterioration in different functional areas and well-being. 32.5% expressed suicidal ideation or suicide attempt.	S	4	C
Walker et al. 2013 USA	Determine whether or not there is a statistically significant relationship between pain severity and life satisfaction in patients with chronic pain.	Cross-sectional study. N=172 people	Pain Severity Scale (PSS) West-Haven-Yale Multidimensional Pain Inventory (WHYMP) Satisfaction with Life Scale (SWL) Coping Responses Inventory-Adult (CRI-A)	A statistically significant negative correlation was observed between pain severity and life satisfaction. The relationship between pain severity and life satisfaction seems to change depending on the level of coping approach exhibited by individuals suffering from chronic pain.	ADL, S, PH	4	C
Wilson et al. 2015 Canada	Describe what adults with chronic pain experience in their role as parents.	Qualitative. In-depth interviews. N=130 people	Brief Pain Inventory (BPI) Numerical Rating Scale (NRS) Pain Catastrophizing Scale-Parent version	Parents with chronic pain are more protective and empathic for children. 81% of parents talk about their experiences of pain with their children, usually when they have to tell them they can’t do anything as a result of pain.	SLFC	5	D
Wing et al. 2016 France	Examine associations between chronic pain and psychiatric morbidity.	Cross-sectional study. N=370 people	7-item Chronic Pain Grade Questionnaire (CPG-7) Numerical Rating Scale (NRS)	Patients with higher pain intensity and interference in social activities were more likely to have depression and anxiety.	S	4	C
Yamada et al. 2016 Japan	Examine the association between psychosocial factors related to work and the prevalence of health-related quality of life.	Cross-sectional study. N=1,764 people	EuroQol-5D (EQ-5D)	The prevalence of pain in workers was higher than in workers. Women had more severe depressive symptoms than men.	SP	4	C

ADL: Activities of daily living; S: sleep; PH: psychological health; SLFC: socio-labor and family consequences; LE: levels of evidence; DR: degree of recommendation.

### Consequences on everyday activities

Chronic Non-Cancer Pain increases in disability and limitation in daily activities^22 - 26^ . People tend to avoid activities that cause or increase the severity of pain, leading to a decrease in activity levels compared with healthy people^22 - 24 , 27^ . In this sense, when pain interferes with the ability to perform daily activities, people tend to show a negative perception of themselves due to lower physical capacity and misunderstanding of people in their environment^28^ , a fact that translates into greater catastrophizing in the face of pain^29^ . In turn, studies indicate that people with CNCP spend more time sitting and less time standing, and that an increase in physical activity during the afternoon is related to the decrease in activities of daily living in the evening^30^ .

A less active lifestyle is also associated with persistence and more severe pain levels^31^ , comorbidity of chronic diseases such as obesity or diabetes^30 , 32^ , more difficulty in self-care and increased use of health services^33^ .

### Consequences of sleep

Sleep problems are a common consequence in people with CNCP^29^ . Studies suggest pain management improve sleep quality by up to 14%. The results indicate there is a circular and dynamic interrelation in which pain causes sleep disorders, which, in turn, increase pain intensity^34 - 36^ .

Other studies state that between 50 and 88% of people meet the diagnostic criteria for sleep disorders^36 , 37^ and have abnormal brain activity during sleep^38^ . In a study conducted by Cranford et al.^37^ , it was reported that 30% of the participants had sleep problems every night and/or several times a night. The most common changes are difficulty in starting or maintaining sleep, waking up earlier, having fragmented or no-restorative sleep^34^ , and higher levels of fatigue during the day; more evident symptoms in women than in men^38^ .

Sleep disorders due to CNCP are also related to limitation in performing daily activities and disability^34^ . Andrews et al.^4^ noted that people performing more intense activities during the day suffer more pain and stay awake longer periods at night.

On the other hand, some people resort to using cannabis to improve the quality of their sleep. Cranford et al.^37^ found that 80% of study participants responded to having used cannabis in the last six months to improve the quality of their sleep, showing a positive opinion about them. However, in 65% of cases people have developed cannabis dependence.

### Consequences on psychological health

CNCP is associated with suffering from mental illness^4^ . Different studies have investigated this relationship, finding that mental illnesses are present in 75.3% of cases of pain^4 , 14^ , and anxiety and depression rates reach 30-40%, being more pronounced in women^13^ . Anxiety and depression cause fear of pain-enhancing activities, generating a spiral of pain, fear and avoidance^24 , 39 - 42^ .

Several authors highlight the relationship between adaptability and CNCP experiences. Thus, people with higher self-esteem have much less unpleasant pain stimuli, improving the use of coping strategies^43 , 44^ . The findings of El-Shormilisy et al.^45^ suggest that coping is also mediated by sex. Women with CNCP develop fewer adaptation strategies than men, resulting in worse functional outcomes.

On the other hand, the prevalence of suicidal ideation and suicide attempts in the population with CNCP is 20% and 5 to 14%, respectively, twice the general population^3 , 45^ . According to Campbell et al.^3^ , this relationship may be due to many people who do not reduce the intensity of their pain, despite undergoing lifelong treatment.

The relationship between CNCP and tobacco use is also complex. According to Orhurhu et al.^14^ , 25.3% of people with CNCP use tobacco, twice the general population. A study by Dirtre et al.^48^ found that 43% of participants who used tobacco also had CNCP. These people reported tobacco use as an agent who calmed anxiety caused by pain. In this sense, the relationship between CNCP and tobacco use is dynamic. Smokers have higher pain rates, increased number of painful sites and higher levels of anxiety and depression, which would encourage tobacco use^14 , 48^ . Thus, pain would strengthen tobacco dependence that results in greater pain^48^ .

### Socio-employment and family consequences

Several studies have shown CNCP causes social isolation, decreased leisure activities and work difficulties^20 , 23 , 47^ . However, people who have greater social participation may have better health, reduced anxiety, depression and reduced perceived stress^23 , 24^ . In this sense, El-Shormilisy et al.^11^ , claim that women and men with CNCP are differently related to their social environment. According to this author, women are socialized from an early age to express their emotions and seek social support, and men would be more likely not to do so. This could translate into greater resources to deal with the CNCP in them.

In the field of research, the existence of CNCP poses a great economic burden for the individual and the system. According to Dany et al.^48^ , most people need to keep working despite the pain, especially in these groups with fewer resources, and they may have less productivity at work.

The family environment of people with CNCP may also be affected^24 , 49^ , as it impacts the family scope, its functioning, and how its members communicate or resolve conflicts. Reactions from other members can trigger different types of invalid responses, rejection, and/or support^22^ . In this sense, difficulties with the partner contribute to increased depression and anxiety^50^ .

Regarding parental relationships, parents model the perceptions and attitudes of their sons and daughters in the face of pain through their own experience, taking as an example the actions and discourses they observe. In turn, pain may affect parents’ ability to complete physical tasks related to creation^49^ .

## DISCUSSION

The increase in scientific production on chronic pain in adulthood allowed us to characterize aspects of interest and detect new lines of research, despite having this Scoping Review as the main limitation of linguistic bias, since there were no studies published in languages other than Spanish or English.

This review shows the ability to perform activities of daily living, sleep, psychological health, labor socio-economic consequences and family functioning are lines of interest in articles published. However, knowledge gaps are detected in areas such as consequences of work, toxic consumption and gender inequalities.

There is an extensive literature on the impact of CNCP on functional capacity, but comorbidity with other chronic diseases is less known. Butchart et al.^51^ , found that about 30% of people with coronary heart disease and chronic obstructive pulmonary disease (COPD) also suffer pain. Torrance et al.^52^ state that comorbidity between chronic pain and one of these two diseases increases by up to three times the probability of dying compared with the population who do not suffer from pain. These authors explain this relationship as a result of the decrease in the daily functioning capacity of people with CNCP. These higher rates of comorbidity, mortality and dependence result in greater use of health services and, therefore, economic expenses, as found by Pitcher et al.^31^ , in our results. However, few studies produce actual figures on the real economic impact of CNCP. Direct and indirect costs in Europe are estimated at 200 million euros and in the US between US$560 million and US$635 million^54^ .

As to psychological health, numerous authors have found that the condition of mental illness, such as anxiety or depression in people with CNCP, is common. Knowing this relationship is important, because authors such as Rayner et al.^55^ report that an effective approach and treatment of depression and anxiety in people with CNCP can improve their health by up to 14%, reducing thus functional limitation and impact on health systems. On the other hand, other authors consider this relationship inverse, that is, chronic pain would be one of the first consequences of mental illness^56 - 59^ . However, for authors such as Von Korff et al.^60^ , the temporal relationship between mental illness and CNCP is unclear, it seems to be bidirectional and both act as a positive reinforcement for the other.

People with CNCP have higher rates of tobacco use. In our results, authors such as Catalano et al.^13^ and Ditre et al.^48^ affirm that tobacco habit increases the level of pain. However, Shi et al.^61^ observe that this relationship has not been shown, but they insist that depressive symptoms associated with tobacco use can increase pain levels.

No studies on the relationship between chronic pain and alcohol consumption were found. We consider it necessary to conduct research on this subject considering the results obtained by Riley & King^62^ in 2009, in which they show that alcohol may be used to relieve pain by its transient analgesic effect. Some results contradict the Danish cohort study by Ekholm et al.^63^ , concluding that patients with CNCP are less likely to consume alcohol, but it is risky when mixed with the drug^64^ .

Regarding labor consequences, the loss of work and the economic cost that generates pain are little studied. Yamada et al.^65^ claim that people with CNCP may have lower productivity rates, which would be a heavy economic burden. In this sense, Pain Proposal’s results support this statement, adding that 21% of the population with CNCP felt unable to finish their working day, which translates into an impact on the labor market of 2.5 million euros and 52 million of lost working days per year^10^ . They also indicate that in Spain, the average number of days lost due to pain is 16.8 days a year^66^ .

Regarding gender inequalities, research shows that, as in other clinical pathologies, women receive more precarious health care than men^67^ . Several studies have found great differences in subjective perception of pain or general well-being in women, despite having scores similar to those of men in analytical and radiological data, which may lead us to think that it is devaluing itself with the use of clinical indices that do not reflect the women’s reality. In addition, the authors emphasize the importance of gender analysis in the study of CNCP^68^ . In this review, the studies found prevalence data disaggregated by sex, and some, in a transversal way, identify differences in male and female experiences, but do not analyze them from a gender perspective^69^ .
