# Supplementary material for: Consequences of Chronic Non-Cancer Pain in adulthood. Scoping Review
Source: Rev Saude Publica. 2020 Apr 3;54:39. doi: 10.11606/s1518-8787.2020054001675 (PMC7135143; doi:10.11606/s1518-8787.2020054001675)
Supplement: Translation

# Translation: Consecuencias del Dolor Crónico no Oncológico en la edad adulta. Scoping Review.

**Authors:** Rocío Cáceres-Matos, Eugenia Gil-García, Sergio Barrientos-Trigo, Ana María Porcel-Gálvez, Andrés Cabrera-León

**Affiliations:** I España Universidad de Sevilla. Facultad de Enfermería, Fisioterapia y Podología. Departamento de Enfermería. Sevilla, España; II España Escuela Andaluza de Salud Pública. Granada, España; III España Centro de Investigación Biomédica en Red de Salud Pública y Epidemiología (CIBERESP). Madrid, España; IV España Universidad de Granada. Instituto de Investigación Biosanitaria, ibs. Hospitales Universitarios de Granada. Granada, España

**Keywords:** Niño, Adolescente, Dolor Crónico, Costo de Enfermedad, Actividades Cotidianas, Calidad de Vida, Revisión

## Abstract

**OBJETIVO:**

Examinar y mapear las consecuencias del dolor crónico en la edad adulta.

**MÉTODO:**

Se incluyeron documentos que abordaban las repercusiones del dolor crónico en las esferas psicológica y social de las personas que lo padecen, publicados en español e inglés entre los años 2013–2018. Aquellos que abordaban tratamientos farmacológicos, dolor crónico derivado de intervenciones quirúrgicas o que no tenían acceso a texto completo fueron excluidos. Finalmente, se incluyeron 28 documentos de los 485 revisados.

**RESULTADOS:**

Los estudios muestran que el dolor se relaciona con altas tasas de limitación en las actividades de la vida diaria, alteraciones del sueño y trastornos del espectro ansiedad-depresión. Las personas con dolor experimentan más problemas para rendir en la jornada laboral y mantener relaciones sociales. Con respecto a la familia, el dolor crónico se ha asociado con un peor funcionamiento familiar.

**CONCLUSIONES:**

Esta revisión pone de manifiesto que las limitaciones en la capacidad para realizar actividades de la vida diaria, el sueño, la salud psicológica, los recursos sociales y laborales y el funcionamiento familiar son líneas de interés en los trabajos publicados. Sin embargo, se detectan lagunas de conocimiento en áreas como la influencia de haber padecido dolor en la infancia o adolescencia, las consecuencias por incumplimiento de la jornada laboral y las desigualdades de género.

## INTRODUCCIÓN

El dolor es una experiencia sensorial y emocional desagradable que actúa como señal biológica de advertencia ante un daño tisular real o potencial^1^ . Se puede clasificar atendiendo a la etiología, localización anatómica o duración^2^ . Según esta última, el dolor crónico no oncológico (DCNO) es aquel que persiste de manera continua o intermitente durante un periodo superior a tres meses y no se asocia a procesos oncológicos^3^ .

El DCNO es considerado un problema de salud pública que afecta al 20–35% de la población mundial^4^ , 19% de la población europea^5^ y al 17% de la población en España^6^ . Cuando el dolor persiste durante periodos de tiempo largos pierde su propósito protector^7^ y se convierte en el resultado de un complejo proceso en el que influyen factores biológicos, psicológicos y socioculturales^8^ en interacción unos con otros^9^ . Distintas organizaciones internacionales como la *European Pain Federation* o la *International Association for the Study of Pain* consideran necesario un cambio de perspectiva en la valoración y tratamiento del dolor. Es preciso abordarlo como una entidad en sí misma^10^ , en la que se debe tener en cuenta el contexto familiar, social y cultural que vive la persona que lo padece^11^ .

En los últimos diez años se ha producido un incremento de la producción científica sobre las repercusiones del dolor. Numerosas investigaciones afirman que en la percepción y valoración del DCNO influyen factores como el sueño^12^ o las limitaciones en la actividad diaria^13^ , pero también factores psicológicos y sociales como la ansiedad, depresión^14^ , autoestima^15^ , afrontamiento^16^ , resiliencia^13^ , apoyo social y familiar^7^ y/o consumo de sustancias tóxicas^14^ .

Por ello, consideramos necesario conocer las consecuencias individuales y sociolaborales que tiene el DCNO en la población adulta. Para ello realizamos una revisión de la literatura científica mediante *Scoping Review* . Nuestro objetivo es examinar y mapear las consecuencias del dolor crónico en la edad adulta.

## MÉTODOS

La metodología de revisión *Scoping Review* se utiliza para mapear la literatura científica y detectar las áreas de estudio que no están suficientemente investigadas^16^ Para llevarla a cabo utilizamos el marco metodológico propuesto por Arksey y O’Malley^17^ y el manual para *Scoping Review* del Joanna Briggs Institute^18^ . Si bien la metodología de la *Scoping Review* no requiere evaluación de la calidad, diversos autores la consideran una fortaleza^19^ ya que permite hacer recomendaciones para la práctica clínica^20^ .

Teniendo en cuenta estas consideraciones, hemos realizado la evaluación del nivel de evidencia (NE) y del grado de recomendación (GR) de los estudios incluidos atendiendo a las pautas recomendadas por la *Scottish Intercollegiate Guidelines Network* ^21^ . En este caso, las puntuaciones NE y GR están relacionadas. Así, a una investigación con NE 1++; 1+ ó 1- se la considera extremadamente recomendable (GR A); para NE 2++, 2+ ó 2- el grado de recomendación sería favorable (GR B), y para NE 3 y 4 los grados de recomendación serían favorables pero no concluyentes (GR C) y no se recomienda ni se desaprueba (GR D) respectivamente.

### Bases de datos y estrategia de búsqueda

La búsqueda en la literatura se realizó en cuatro bases de datos científicas (CINAHL, PubMed, Scopus y Web of Science) y en depósitos de literatura gris (Depósito de Investigación de la Universidad de Sevilla IDUS, TESEO, Openthesis, Opengrey, Grey Literature Report, *American Pain Society, European Pain Federation* e *International Association for the Study of Pain* ).

Se llevaron a cabo entre noviembre y diciembre de 2018 con la siguiente estrategia de búsqueda: (“chronic pain” OR “persistent pain” OR “long term pain”) and (adult*) AND (impact* OR influenc* OR cause* OR outcome* OR result* OR consequence* OR effect* OR repercussion*).

Como criterios de inclusión se consideraron: 1) documentos que abordaban las consecuencias biopsicosociales que genera el dolor crónico no oncológico; 2) en personas adultas entre 18 y 65 años, no hospitalizadas; 3) publicados entre 2013 y diciembre de 2018 y 4) escritos en español o inglés.

Como criterios de exclusión fueron usados: 1) documentos que abordaran tratamientos farmacológicos; 2) documentos que abordaran personas con dolor crónico no oncológico derivado de enfermedades neurodegenerativas, infecciosas de carácter crónico o deterioro cognitivo y 3) documentos que no dispusieran de acceso al texto completo.

### Cribado

Tras la eliminación de los artículos duplicados en la búsqueda, se realizó el proceso de selección, evaluando la relevancia de los estudios identificados. Este proceso lo llevaron a cabo dos revisores de manera independiente.

En la fase primera, los revisores evaluaron los títulos y resúmenes encontrados para excluir los artículos que no cumplían con los criterios de inclusión y exclusión. Posteriormente, revisaron su contenido a texto completo y decidieron cuáles fueron incluidos en la fase de extracción de los datos. En caso de desacuerdo en la inclusión de un artículo entre los dos revisores, se discutió con el resto del equipo de investigación hasta llegar a un consenso.

### Extracción y análisis de datos

Los miembros del equipo elaboraron un documento que sirvió como modelo para que la extracción de la información de los artículos estuviese estandarizada. Además, desarrollaron un protocolo para sistematizar el procedimiento de trabajo. Dos de los miembros del equipo recogieron de manera independiente información acerca de cada uno de los artículos (nombre de los autores, año y país de publicación, objetivo y metodología del estudio, tamaño y características de la muestra, intervención, principales resultados obtenidos y puntos clave). Todos los miembros del equipo discutieron la información en los casos de desacuerdo hasta llegar al consenso.

## RESULTADOS

Tras la revisión, se obtuvieron 485 artículos cuyos títulos cumplían los criterios de inclusión. La búsqueda de la literatura gris produjo 60 documentos. Una vez eliminados los duplicados, se procedió a la revisión de 285 títulos y resúmenes utilizando los criterios de inclusión y exclusión. Se recuperaron 170 textos completos para el cribado. Tras la revisión de los textos completos, 142 fueron excluidos, ya que no informaban acerca del tema de estudio, quedando finalmente 28 documentos para su análisis ( Figura ). Los estudios incluidos se clasificaron en cuatro áreas en función de las afectaciones del dolor crónico: actividades de la vida diaria (AVD); sueño (S); salud psicológica (SP) y consecuencias socio-laborales y familiares (CSLF) ( Tabla ).

**Figura:** Consecuencias del dolor crónico no oncológico en la edad adulta. *Scoping Review* .

**Tabla:** Características principales de los estudios incluidos en la revisión.

Autores, año, país	Objetivo	Metodología/muestra	Intervención	Resultados	Puntos clave	NE	GR
Altug et al. 2015 Turquía	Comparar el dolor, el estado emocional y el nivel de discapacidad en pacientes con dolor cervical y dolor lumbar crónicos.	Estudio transversal. N=100 pacientes	Visual Analogue Scale (VAS) Owestry Disability Index (ODI) Neck Disability Index (NDI) eck Depression Inventory (BDI)	No se encontró diferencia significativa para la intensidad y duración del dolor entre el grupo de lumbalgia y el de dolor de cuello. La duración del dolor, el estado emocional y el nivel de discapacidad en pacientes con dolor lumbar fueron peores que en los pacientes con dolor de cuello.	S, SP	4	C
Akbari et al. 2016 Irán	Examinar el papel de las variables individuales y relacionadas con la familia en la comprensión del dolor crónico relacionado con la discapacidad.	Estudio transversal. N=142 personas	Visual Analogue Scale (VAS) Tampa Scale of Kinesiophobia (TSK) Pain Catastrophizing Scale (PCS) Roland Morris Disability Questionnaire (RDQ) Anxiety Stress Scales (DASS) Family Assessment Device (FAD)	La disfunción familiar puede contribuir al pensamiento catastrófico, que a su vez contribuye a la discapacidad de los pacientes aumentando el temor a movimiento y depresión.	S, SP, CSLF	4	C
Andrews et al. 2014 Australia	Examinar la asociación entre la actividad física diaria y el sueño en personas con dolor crónico.	Estudio observacional prospectivo. N= 50 personas	Cuestionario demográfico Visual Analogue Scale (VAS)	Los individuos que afirmaron tener un mayor número de puntos de dolor tenían períodos más largos de vigilia por la noche.	AVD	4	C
Bailly et al. 2015 Francia	Comprender las experiencias de los pacientes que viven con dolor de espalda baja.	Estudio cualitativo monocéntrico. N=25 personas (11 hombres y 14 mujeres)	Entrevista semiestructurada y grupos focales	Los participantes refirieron una percepción negativa en las interacciones sociales, con vergüenza y frustración con respecto a sus dificultades para realizar actividades de la vida diaria. Se sentían incomprendidos y sin apoyo debido a la ausencia de signos visibles de la condición.	SP	5	D
Boggero et al. 2015 USA	Probar las contribuciones independientes e interactivas del componente somatosensorial del dolor y el componente afectivo del dolor en el funcionamiento emocional, social y diario.	Estudio transversal N=472 personas	The West Haven-Yale Multidimensional Pain Inventory (MPI) Visual Analog Scale (VAS)	Las mujeres mostraron una actividad general más alta que los varones. Mayores niveles de dolor se tradujeron en peores resultados en todas las áreas de funcionamiento.	S, SP	4	C
Cabrera-Perona et al. 2017 España	Evaluar la asociación entre la comparación social, catastrofismo y resultados de salud específicos.	Estudio transversal. N= 131 personas		Se encontró correlación positiva entre el catastrofismo y la incapacidad funcional, angustia psicológica, ansiedad y depresión en fibromialgia.	S, SP	4	C
Calandre et al. 2015 España	Evaluar la prevalencia de la ideación suicida entre una muestra de pacientes con fibromialgia.	Estudio transversal. N=373 personas	Fibromyalgia Impact Questionnaire The Beck Depression Inventory (BDI) The Pittsburgh Sleep Quality Index The Brief Pain Inventory (BPI) SF-12 Health Survey	El 48% de las personas con dolor crónico refirieron haber tenido pensamientos suicidas y el 31% había intentado suicidarse. No había diferencias en la edad o duración de la enfermedad entre los pacientes sin ideación suicida, pensamiento suicida y conducta suicida.	S	4	C
Campbell et al. 2013 UK	Probar que los problemas de sueño en las personas con dolor crónico aumentan el riesgo iniciar depresión.	Estudio prospectivo de cohortes. N=2.622 personas	Jenkins Sleep Questionnaire Hospital Anxiety and Depression Scale (HADS)	Los problemas de sueño pueden generar depresión tres años después de su inicio. Los tratamientos pueden ser prevenir o tratar los problemas para dormir, además de enfocarse en el manejo del dolor.	AVD, S	1b	A
Campbell et al. 2015 Nueva Zelanda	Documentar la prevalencia y correlatos del dolor crónico y el suicidio, estimando la contribución de la enfermedad crónica a la suicidalidad.	Estudio poblacional. N=8.841 personas.	Encuesta Nacional Australiana de Salud Mental.	Las probabilidades suicidio fueron de dos a tres veces mayores en personas con dolor. El 65% de las personas que intentaron suicidarse en los últimos 12 meses tenían antecedentes de dolor crónico.	S	3b	B
Castro et al. 2014 España	Evaluar la calidad del sueño y la prevalencia del trastorno depresivo mayor y el trastorno de ansiedad generalizada en pacientes con dolor crónico osteomuscular.	Estudio transversal. N= 39 personas	Entrevista Neuropsiquiátrica Internacional Pittsburgh Questionnaire Montgomery-Asberg Depression Scale	El 69,2 % de la muestra presentaba un trastorno depresivo mayor y/o un 65,5 % un trastorno de ansiedad generalizada. No se encontraron diferencias estadísticamente significativas en la calidad del sueño entre los grupos tratados con opioides potentes y tratados con opioides menores/antiinflamatorios.	AVD, S	4	C
Danya et al. 2015 Francia	Explorar la relación entre la perspectiva de tiempo y el distrés psicológico.	Estudio transversal N=264 personas	Zimbardo Time Perspective Inventory (ZTPI) Pain Beliefs and Perceptions Inventory (PBPI) Hospital Anxiety and Depression Scale (HADS)	Los pacientes con hijos estaban más deprimidos, aquellos con nivel educativo eran más ansiosos y aquellos que estaban desempleados durante su enfermedad tenían niveles de depresión más altos.	S, SP	4	C
De Sola et al. 2017 España	Determinar la prevalencia y los factores relacionados con los permisos por enfermedad y la pérdida de trabajo entre personas que sufren dolor crónico.	Estudio transversal. N=1.543 personas	ncuesta telefónica	La prevalencia de baja por enfermedad debido a CP en la población española fue del 4.21%, siendo más prevalente en personas necesitan ayuda para vestirse y asearse, tomar medicamentos y/o con mayor nivel educacional. La prevalencia de pérdida de empleo fue del 1.8%.	SP, CSLF	4	C
Ditre et al. 2013 USA	Evaluar la importancia de la ansiedad relacionada con el dolor en relación con la dependencia del tabaco en una muestra de fumadores con dolor crónico.	Estudio transversal. N=129 personas	Pain Anxiety Symptoms Scale (PASS-20) Wisconsin Inventory of Smoking Dependence Motives (WISDM) Chronic Pain Screener Graded Chronic Pain Scale (GCPS) Generalized Anxiety Disorder (GAD-7) Fagerström Test for Nicotine Dependence (FTND)	La ansiedad relacionada con el dolor estaba fuertemente asociada con la dependencia al tabaco. Los fumadores de tabaco con dolor pueden estar en riesgo de mantener y exacerbar su dependencia del tabaco, posiblemente debido a las diferencias individuales en la ansiedad relacionada con el dolor.	S	4	C
Ferreira-Valente et al. 2014 Portugal	Evaluar las respuestas de afrontamiento, creencias y apoyo social para adaptarse positivamente	Estudio observacional. N= 324 personas	Numerical Rating Scale (NRS) Portuguese Brief Pain Inventory Interference Scale (P-BPI) Physical Component Summary (MCS) Portuguese Pain Self-Efficacy Questionnaire (P-PSEQ) Social Support Satisfaction Scale (ESSS) Portuguese 2-item per Scale Coping Strategies Questionnaire (CSQ-14)	Las respuestas de afrontamiento y el apoyo social percibido se relacionaron con la interferencia del dolor y el funcionamiento psicológico. Las respuestas de afrontamiento se asociaron positivamente con la interferencia del dolor y negativamente con el funcionamiento físico y psicológico.	AVD, S, SP	4	C
Henne et al. 2015 Australia	Explorar la relación entre el dolor persistente, angustia y conexión emocional.	Estudio transversal. N=388 mujeres	The McGill Pain Questionnaire (SF-MPQ) Depression, anxiety and stress (DASS-21– short form)	Muchas mujeres con dolor crónico presentan dificultades de conexión emocional que atribuyen a la experiencia de dolor persistente.	S	4	C
Inoue et al. 2015 Japón	Estimar la prevalencia del dolor crónico en Japón, analizar los factores asociados y evaluar la carga social debida al dolor crónico.	Estudio transversal N=6.000 personas	11-point Numerical Rating Scale (NRS)	El dolor crónico empeora con factores ambientales. Las personas que realizan ejercicio físico tuvieron menores tasas de dolor. Las mujeres sufrieron más dolor que los hombres. La edad avanzada, vivir solo y estar desempleado también se asoció con el dolor crónico.	SP	4	C
Jensen et al. 2015 Dinamarca	Describir las asociaciones entre la demografía y la calidad de vida relacionada con la salud de los pacientes con dolor crónico no oncológico.	Estudio de cohortes N=1.176 personas	Hospital Anxiety and Depression Scale (HADS) Short-Form 36 Bodily Pain Scale (SF-36).	Los pacientes con dolor crónico obtuvieron puntuaciones más bajas en todos los dominios, especialmente en la actividad física, nivel de dolor y vitalidad. El 75% de los pacientes evalúan que su salud física afecta a su trabajo y/o actividad diaria.	SP	1b	A
Karaman et al. 2014 Turquía	Examinar la prevalencia y la relación entre los trastornos del sueño y el dolor crónico.	Estudio observacional. N=2.635 personas	Visual Analogue Scale (VAS) Pain Sleep Quality Index (PSQI)	Un 33% de los pacientes con dolor crónico en las edades entre 20-30 años y 47.8% entre 60-70 años tenían peor calidad en el sueño.	AVD	4	C
Mun et al. 2017 USA	Probar el efecto de la expectativa del dolor con el dolor posterior.	Estudio transversal. N= 451 personas	Cumplimentación de un diario de dolor.	El control del dolor hoy predice la disminución del dolor en el día siguiente. La negatividad y el afrontamiento no se relacionan con el nivel de dolor al día siguiente.	SP	4	C
Nicholas et al. 2017 USA	Examinar la interacción entre el dolor y el funcionamiento físico a corto plazo.	Estudio longitudinal. N=389 personas	15-item Brief Pain Inventory 2-item Short-Form 36 Bodily Pain Scale (SF-36)	Las terapias para controlar las consecuencias del dolor crónico en aspectos relevantes de la vida y de la realización de actividades físicas son necesarias para la restauración de la actividad vital.	SP	2b	B
Orhurhu et al. 2015 USA	Determinar la prevalencia del tabaquismo en pacientes con dolor crónico.	Estudio poblacional N=5.350 pacientes		La prevalencia del tabaquismo en adultos con dolor crónico fue del 23.5%.	S	4	C
Raijmakers et al. 2015 Alemannia	Determinar las diferencias en el nivel de actividad diaria entre personas con y sin dolor.	Ensayo clínico N=37 personas		Los pacientes con dolor crónico presentan un nivel general de actividad física menor que la población sana.	SP	1b	A
Stefane et al. 2013 Brasil	Evaluar el dolor percibido, la discapacidad y la calidad de vida en individuos con dolor lumbar crónico.	Estudio transversal. N=97 personas	Roland Morris Questionnaire (RMQ) WHOQOL-Brief	El puntaje de intensidad de dolor percibido, la discapacidad y la calidad de vida fueron los más afectados.	AVD, SP	4	C
Triñanes et al. 2014 España	Describir la relación de la ideación suicida en fibromialgia.	Estudio transversal. N=117 mujeres	Beck Depression Inventory (BDI) The Pittsburgh Sleep Quality Index (PSQI) The Fibromyalgia Impact Questionnaire (FIQ) Short-Form 36 Health Survey (SF-36) Visual Analogue Scale (VAS)	Los pacientes con fibromialgia presentan un nivel moderado de depresión, disfunción del sueño y deterioro severo en diferentes áreas funcionales y bienestar. El 32.5% manifestó ideación o intento suicida.	S	4	C
Walker et al. 2013 USA	Determinar si existe o no una relación estadísticamente significativa entre gravedad del dolor y satisfacción con la vida en pacientes con dolor crónico.	Estudio transversal. N=172 personas	Pain Severity Scale (PSS) West-Haven-Yale Multidimensional Pain Inventory (WHYMP) Satisfaction with Life Scale (SWL) Coping Responses Inventory-Adult (CRI-A)	Se observó una correlación negativa estadísticamente significativa entre la gravedad del dolor y la satisfacción con la vida. La relación entre la severidad del dolor y la satisfacción con la vida parece cambiar en función del nivel de afrontamiento de aproximación exhibido por individuos que sufren de dolor crónico.	AVD, S, SP	4	C
Wilson et al. 2015 Canadá	Describir lo que los adultos con dolor crónico experimentan en su rol como padres.	Cualitativo. Entrevistas en profundidad. N=130 personas	Brief Pain Inventory (BPI) Numerical Rating Scale (NRS) Pain Catastrophizing Scale-Parent version	Los padres con dolor crónico son más protectores y empáticos con los hijos. El 81% de los padres hablan sobre sus experiencias de dolor con sus hijos, normalmente cuando tienen que decirles que no pueden hacer algo a consecuencia del dolor.	CSLF	5	D
Wing et al. 2016 Francia	Examinar las asociaciones entre el dolor crónico y la morbilidad psiquiátrica.	Estudio transversal N=370 personas	7-item Chronic Pain Grade Questionnaire (CPG-7) Numerical Rating Scale (NRS)	Los pacientes con mayor intensidad de dolor e interferencia en las actividades sociales, tuvieron más probabilidades de depresión y ansiedad.	S	4	C
Yamada et al. 2016 Japón	Examinar la asociación entre los factores psicosociales relacionados con el trabajo y la prevalencia de la calidad de vida relacionada con la salud.	Estudio transversal N=1.764 personas	EuroQol-5D (EQ-5D)	La prevalencia de dolor en las trabajadoras fue mayor que en los trabajadores. Las mujeres presentaron más síntomas depresivos severos que los hombres.	SP	4	C

AVD: Actividades de la vida diaria; S: sueño; SP: salud psicológica; CSLF: consecuencias socio-laborales y familiares; NE: niveles de evidencia; GR: grado de recomendación.

### Consecuencias sobre las actividades de la vida diaria

El Dolor Crónico no Oncológico ocasiona un aumento de la discapacidad y limitación en la actividad diaria^22^ . Las personas tienden a evitar actividades que causen o aumenten la severidad del dolor, lo que lleva a una disminución en los niveles de actividad en comparación con las personas sanas^22^ . En este sentido, cuando el dolor interfiere en la capacidad para realizar las actividades de la vida diaria, las personas tienden a mostrar una percepción negativa de sí mismas debido a la menor capacidad física y a la incomprensión de las personas de su entorno^28^ , hecho que se traduce en mayor catastrofismo ante el dolor^29^ . A su vez, los estudios indican que las personas con DCNO pasan más tiempo sentadas y menos de pie, y que un aumento de la actividad física durante la tarde se relaciona con disminución de las actividades de la vida diaria durante la noche^30^ .

Un estilo de vida menos activo se asocia también con persistencia y niveles de dolor más severos^31^ , la comorbilidad de enfermedades crónicas como obesidad o diabetes^30 , 32^ , más dificultad para el autocuidado y mayor uso de servicios sanitarios^33^ .

### Consecuencias sobre el sueño

Los problemas del sueño son una consecuencia común en las personas con DCNO^29^ . Los estudios apuntan que el control del dolor mejoraría la calidad del sueño hasta en un 14%. Los resultados indican que existe una interrelación circular y dinámica en la que el dolor ocasiona trastornos del sueño y los trastornos del sueño, a su vez, incrementan la intensidad del dolor^34^ .

Otros estudios afirman que entre un 50–88% de las personas cumplen los criterios diagnósticos de trastornos del sueño^36 , 37^ y presentan una actividad cerebral anormal durante el sueño^38^ . En un estudio de Cranford et al.^37^ refirieron que el 30% de los participantes indicaron que sus problemas del sueño ocurrían todas las noches y/o varias veces por noche. Las alteraciones más frecuentes son dificultad para la iniciación o mantenimiento del sueño, despertares tempranos, sueño fragmentado o no reparador^34^ , y mayores niveles de fatiga durante el día; síntomas más acusados en mujeres que en hombres^38^ .

Las perturbaciones del sueño debido al DCNO se relacionan también con la limitación en la realización de actividades de la vida diaria y la discapacidad^34^ . Andrews et al.^4^ , señalaron que las personas que realizan actividades de mayor intensidad durante el día padecen mayor número de puntos dolorosos y periodos de vigilia más largos durante la noche.

Por otro lado, algunas personas recurren al consumo de cannabis para mejorar la calidad de su sueño. Cranford et al.^37^ , encontraron que el 80% de los participantes de su estudio respondieron haber consumido cannabis en los últimos seis meses para mejorar la calidad de su sueño, mostrando una opinión positiva sobre ellos. Sin embargo, en el 65% de los casos las personas desarrollaron dependencia al cannabis.

### Consecuencias sobre la salud psicológica

El padecimiento del DCNO se asocia con la comorbilidad de enfermedades mentales^4^ . Diferentes estudios han investigado esta relación encontrando que las enfermedades mentales están presentes en el 75,3% de los casos de dolor^4 , 14^ y las tasas de ansiedad y depresión alcanzan el 30–40%, siendo más acusado en mujeres^13^ . La ansiedad y la depresión ocasionan temor ante las actividades que aumentan el dolor, generando una espiral de dolor, miedo y evitación^24 , 39^ .

Diversos autores ponen de manifiesto la relación entre la capacidad de adaptación y las vivencias del DCNO. Así, las personas con mayor autoestima presentan estímulos de dolor mucho menos desagradables, mejorando el uso de estrategias de afrontamiento^43 , 44^ . Los hallazgos de El-Shormilisy et al.^45^ , sugieren que el afrontamiento también está mediado por el género. Las mujeres con DCNO desarrollan menos estrategias de adaptación que los hombres, lo que conlleva a peores resultados funcionales.

Por otro lado, la prevalencia de ideación suicida e intentos de suicidio en la población con DCNO es del 20% y 5–14% respectivamente, el doble que en la población general^3 , 45^ . Según Campbell et al.^3^ , esta relación puede deberse a que muchas personas no logran la disminución de la intensidad de su dolor a pesar de seguir un tratamiento de por vida.

La relación entre el DCNO y el consumo de tabaco también es compleja. Según Orhurhu et al.^14^ , el 25,3% de las personas con DCNO consumen tabaco, el doble que en la población general. En un estudio de Dirtre et al.^48^ , encontraron que el 43% de los participantes consumidores de tabaco padecían también DCNO. Estas personas refirieron utilizar el tabaco como un agente que calmaba la ansiedad que les causaba el dolor. En este sentido, la relación entre el DCNO y el consumo de tabaco es dinámica. Los fumadores padecen tasas de dolor más elevadas, mayor número de localizaciones dolorosas y mayores niveles de ansiedad y depresión, lo que favorecería el consumo de tabaco^14 , 48^ . Así pues, el dolor actuaría como un reforzador de la dependencia tabáquica que se traduce en mayor dolor^48^ .

### Consecuencias socio-laborales y familiares

Diversos estudios han manifestado que el DCNO provoca aislamiento social, disminución de las actividades de ocio y dificultades laborales^20 , 23 , 47^ . Sin embargo, las personas que tienen mayor participación social pueden tener un mejor estado de salud, reducción de la ansiedad, depresión y menor estrés percibido^23 , 24^ . En este sentido, El-Shormilisy et al.^11^ , afirman que las mujeres y hombres con DCNO se relacionan de manera diferente con su entorno social. Según este autor, las mujeres son socializadas desde una edad temprana para expresar sus emociones y buscar apoyo social, y los hombres sería más probable que no lo hicieran. Esto podría traducirse en mayores recursos para afrontar el DCNO en ellas.

En el ámbito laboral, la existencia de DCNO supone una gran carga económica para la persona y para el sistema. Según Dany et al.^48^ , la mayoría de las personas necesitan continuar trabajando a pesar del dolor, especialmente en aquellos grupos con menos recursos, siendo probable que tengan una menor productividad laboral.

El entorno familiar de las personas que padecen DCNO también puede verse afectado^24 , 49^ , ya que impacta en la atmósfera familiar, su funcionamiento y en la forma en la que sus miembros se comunican o resuelven conflictos. Las reacciones de los demás miembros pueden desencadenar diferentes tipos de respuestas invalidantes, de rechazo y/o de apoyo^22^ . En este sentido, las dificultades con la pareja contribuyen al aumento de depresión y ansiedad^50^ .

En cuanto a las relaciones parentales, los progenitores modelan las percepciones y actitudes de los hijos e hijas ante el dolor a través de su propia experiencia tomando como ejemplo las acciones y discursos que observan. A su vez, el dolor puede afectar a la capacidad de los progenitores para completar tareas físicas relacionadas con la crianza^49^ .

## DISCUSIÓN

El aumento de la producción científica sobre el dolor crónico en la edad adulta nos ha permitido caracterizar los aspectos de interés y detectar nuevas líneas de investigación a pesar de tener esta *Scoping Review* el como principal limitación el sesgo de idioma, ya que no se incluyeron estudios publicados en idiomas diferentes al español o inglés.

Esta revisión pone de manifiesto que la capacidad para realizar actividades de la vida diaria, el sueño, la salud psicológica, las consecuencias socio-laborales y el funcionamiento familiar son líneas de interés en los trabajos publicados. Sin embargo, se detectan lagunas de conocimiento en áreas como las consecuencias laborales, el consumo de tóxicos y las desigualdades de género.

Existe amplia literatura acerca de las repercusiones que produce el DCNO en la capacidad funcional, pero la comorbilidad con otras enfermedades crónicas es menos conocida. Butchart et al.^51^ , encontraron que alrededor de un 30% de las personas con patologías coronarias y enfermedad pulmonar obstructiva crónica (EPOC) padecen también dolor. Torrance et al.^52^ , afirman que la comorbilidad entre el dolor crónico y una de estas dos enfermedades aumenta hasta en tres la probabilidad de morir con respecto a la población que no padece dolor. Estos autores explican esta relación como el resultado de la disminución de la capacidad para el funcionamiento diario de las personas con DCNO. Estas mayores tasas de comorbilidad, mortalidad y dependencia ocasionan un mayor uso de los servicios sanitarios y por ende de gasto económico, tal y como hace referencia Pitcher et al.^31^ , en nuestros resultados. Sin embargo, existen pocos estudios que arrojen cifras reales sobre el impacto económico real del DCNO. Se estima que los costes directos e indirectos en Europa rondan los 200 millones de euros^53^ y en EE.UU. entre los 560 y los 635 millones de dólares^54^ .

En cuanto a la salud psicológica, numerosos autores han encontrado que el padecimiento de enfermedades mentales, tales como la ansiedad o depresión en personas con DCNO, es frecuente. Conocer esta relación es importante ya que autores como Rayner et al.^55^ , refieren que un abordaje y tratamiento eficaz de la depresión y ansiedad en las personas con DCNO puede mejorar su salud hasta en un 14%, disminuyendo a su vez la limitación funcional y el impacto en los sistemas de salud. Por otro lado, otros autores consideran que esta relación es inversa, es decir, el dolor crónico sería una de las primeras consecuencias de las enfermedades mentales^56^ . Sin embargo, para autores como Von Korff et al.^60^ , la relación temporal entre las enfermedades mentales y el DCNO no está clara, parece ser bidireccional y ambas actúan como reforzador positivo de la otra.

Las personas con DCNO presentan mayores tasas de consumo de tabaco. En nuestros resultados, autores como Catalano et al.^13^ , y Ditre et al.^48^ , afirman que el hábito tabáquico aumenta el nivel de dolor. Sin embargo, Shi et al.^61^ , refieren que esta relación no se ha demostrado pero insisten que en que los síntomas depresivos asociados al consumo de tabaco sí podrían aumentar los niveles de dolor.

No se han encontrado estudios que aborden la relación entre el padecimiento de dolor crónico y el consumo de alcohol. Consideramos necesario realizar investigaciones al respecto teniendo en cuenta los resultados obtenidos por Riley & King^62^ en 2009 en los que ponen de manifiesto que el alcohol se puede estar utilizando para paliar el dolor por su efecto analgésico transitorio. Resultados que contradicen el estudio de cohortes danés de Ekholm et al.^63^ , al concluir que los pacientes con DCNO tienen menos probabilidad de consumir alcohol, pero existen riesgos derivados de su consumo al mezclarlo con medicamentos^64^ .

Respecto a las consecuencias laborales, la pérdida de trabajo y el coste económico que genera el dolor están poco estudiados. Yamada et al.^65^ , afirman que las personas con DCNO pueden mostrar menores tasas de productividad, lo que supondría una gran carga económica. En este sentido, los resultados de la encuesta *Pain Proposal* apoyan esta afirmación, añadiendo además que el 21% de la población con DCNO se sentía incapaz de finalizar su jornada laboral, lo que se traduce en un impacto en el mercado laboral de 2.500 millones de euros y 52 millones de jornadas laborales perdidas al año^10^ . Asimismo indican que, en España, el promedio de días perdidos por dolor es de 16.8 jornadas al año^66^ .

En relación con las desigualdades de género, las investigaciones ponen de manifiesto que, tal y como ocurre en otras patologías clínicas, las mujeres reciben una atención en salud más deficiente que los hombres^67^ . Varios estudios han detectado grandes diferencias en la percepción subjetiva del dolor o bienestar general en las mujeres a pesar de presentar puntuaciones similares a los hombres en los datos analíticos y radiológicos, lo que nos puede llevar a pensar que se está infravalorando con el uso de índices clínicos que no reflejan la realidad de las mujeres. Además, los autores insisten en la importancia del análisis de género en el estudio del DCNO^68^ . En esta revisión, los estudios encontrados presentan datos de prevalencia desagregados por sexos, y algunos, de manera transversal, identifican diferencias en las vivencias de hombres y mujeres, pero no lo analizan desde una perspectiva de género^69^ .

Como líneas prospectivas, sería conveniente profundizar en las consecuencias del dolor crónico en el adulto mayor. Esto permitiría, tras haber estudiado las consecuencias en la infancia y adolescencia^70^ y en la edad adulta, mapear todas las repercusiones que el DCNO genera en las personas que lo padecen.
